# Mortality reduction with use of oral beta-blockers in patients with acute coronary syndrome

**DOI:** 10.6061/clinics/2016(11)03

**Published:** 2016-11

**Authors:** Alexandre de Matos Soeiro, Pedro Gabriel Melo de Barros e Silva, Eduardo Alberto de Castro Roque, Aline Siqueira Bossa, Cindel Nogueira Zullino, Sheila Aparecida Simões, Mariana Yumi Okada, Tatiana de Carvalho Andreucci Torres Leal, Maria Carolina Feres de Almeida Soeiro, Carlos V. Serrano, Múcio Tavares Oliveira

**Affiliations:** I Faculdade de Medicina da Universidade de São Paulo, Instituto do Coração (InCor), Unidade de Emergência, São Paulo/SP, Brazil; II Hospital TotalCor, São Paulo/SP, Brazil; III Hospital Metropolitano, Serra/ES, Brazil

**Keywords:** Acute Coronary Syndromes, Prognosis, Beta-blockers

## Abstract

**OBJECTIVES:**

Recent studies have revealed a relationship between beta-blocker use and worse prognosis in acute coronary syndrome, mainly due to a higher incidence of cardiogenic shock. However, the relevance of this relationship in the reperfusion era is unknown. The aim of this study was to analyze the outcomes of patients with acute coronary syndrome that started oral beta-blockers within the first 24 hours of hospital admission (group I) compared to patients who did not use oral beta-blockers in this timeframe (group II).

**METHODS:**

This was an observational, retrospective and multicentric study with 2,553 patients (2,212 in group I and 341 in group II). Data regarding demographic characteristics, coronary treatment and medication use in the hospital were obtained. The primary endpoint was in-hospital all-cause mortality. The groups were compared by ANOVA and the chi-square test. Multivariate analysis was conducted by logistic regression and results were considered significant when *p*<0.05.

**RESULTS:**

Significant differences were observed between the groups in the use of angiotensin-converting enzyme inhibitors, enoxaparin, and statins; creatinine levels; ejection fraction; tabagism; age; and previous coronary artery bypass graft. Significant differences were also observed between the groups in mortality (2.67% *vs* 9.09%, OR=0.35, *p*=0.02) and major adverse cardiovascular events (11% *vs* 29.5%, OR=4.55, *p*=0.02).

**CONCLUSIONS:**

Patients with acute coronary syndrome who underwent early intervention with oral beta-blockers during the first 24 hours of hospital admission had a lower in-hospital death rate and experienced fewer major adverse cardiovascular events with no increase in cardiogenic shock or sustained ventricular arrhythmias compared to patients who did not receive oral beta-blockers within this timeframe.

## INTRODUCTION

Beta-blockers are the main drug treatment used for patients with acute myocardial infarction (AMI). American Heart Association (ACCF/AHA) guidelines provide a class I recommendation for oral beta-blockers within the first 24 hours of symptom onset and a class IIa indication for intravenous beta-blockers for patients who are hypertensive or have ongoing ischemia 1,2. Not surprisingly, healthcare organizations have adopted the use of beta-blockers at discharge following myocardial infarction as a quality indicator 3-6.

All guidelines that support the use of beta-blockers in AMI predate reperfusion and contemporary medical therapy with statins and antiplatelet agents 3. Recent data have called into question the role of beta-blockers in AMI, particularly regarding the type, dosage and duration of treatment for patients whose post-AMI course is without arrhythmia, heart failure or recurrent ischemia. In the pre-reperfusion era, reductions in mortality have been reported with beta-blocker use, but the role of this treatment in the reperfusion era is not clear. Notably, in the reperfusion era, only reductions in myocardial infarction and angina have been observed, whereas increases in heart failure and cardiogenic shock have occurred 3,7-10.

Based on the above, the objective of this study was to analyze the short-term outcomes of patients with acute coronary syndrome (ACS) who started oral beta-blockers during the first 24 hours of hospital admission and to compare these outcomes to those of patients who did not use oral beta-blockers within this timeframe.

## MATERIALS AND METHODS

### Study population

This study had an observational, multicentric and retrospective design. Databank analysis was performed for 2,553 patients with ACS (ST elevation myocardial infarction [STEMI] and non-ST elevation myocardial infarction [NSTEMI]) who were treated in a tertiary health center between May 2010 and May 2014. The patients were divided into the following two groups: patients who started oral beta-blockers within the first 24 hours of admission (group I, n=2,212) and patients who did not take oral beta-blockers during this timeframe (group II, n=341). Patients who were treated with intravenous beta-blockers or who presented with cardiogenic shock or bradycardia were excluded.

Patients were diagnosed and treated according to AHA/ESC Task Force guidelines 1,2. All patients underwent percutaneous coronary intervention less than 24 hours after the onset of ACS. The following beta-blockers were used: propranolol, atenolol, carvedilol and metoprolol.

The primary outcome was in-hospital all-cause mortality. The secondary outcome was major adverse cardiac events (MACE), including all causes of death, non-fatal angina or AMI/target vessel revascularization, cardiogenic shock, bleeding (major and minor), sustained ventricular arrhythmia and stroke.

The study was approved by the ethics and research committee, and informed consent was obtained from all patients or their family members.

### Analytical methods

The following data were obtained: age, sex, diabetes, systemic arterial hypertension, tabagism, dyslipidemia, familial history of premature coronary artery disease, heart failure, previous coronary artery disease, previous stroke, hematocrit, creatinine, troponin, systolic arterial pressure, left ventricle ejection fraction and medications used within the first 24 hours of admission (Table 1).

Blood was sampled immediately after admission and prior to administration of medications (baseline) and then daily according to institutional protocols. Cardiac markers, such as troponin-I, were measured using standard clinical chemistry. The laboratory upper limits of normal were 0.04 ng/ml (99^th^ percentile) for troponin-I, which was measured using an Elecsys 2010 (Siemens Healthcare Diagnostics Inc., United States of America) 4^th^ generation immunoassay.

Major bleeding was defined using BARC 11 scores: types 3 and 5 indicated major bleeding, while types 1 and 2 indicated minor bleeding. Post-operative bleeding was not considered.

Sustained ventricular arrhythmia was defined as sustained ventricular tachycardia with or without pulse and ventricular fibrillation.

### Statistical analysis

The collected data were submitted to descriptive analysis, including determination of the median and the minimum and maximum values. Comparisons between groups were made by ANOVA and the chi-square test. If the Kolmogorov-Smirnov test confirmed a normal distribution, then continuous variables were summarized using the mean±standard deviation and were compared using Student’s t-test for independent samples. The Mann-Whitney U test was used to compare continuous variables if they were not normally distributed.

Multivariate analysis was performed using logistic regression, and the results were considered significant when *p*<0.05. The variables included all baseline characteristics shown in Table 1.

All statistical procedures were performed using the statistical software package SPSS, v10.0.

## RESULTS

The median age was 62 years, and approximately 40.5% of the cohort was male. The most prevalent risk factor was hypertension (76%), followed by dyslipidemia (48%). In total, 25% of the patients in group I had STEMI compared to 29% of the patients in group II. Approximately 40% of the patients in group I underwent percutaneous coronary intervention compared with 29% of the patients in group II. Coronary artery bypass surgery was performed on 17% of the patients in group I and 8% of the patients in group II.

We observed significant differences in the prevalence of the use of angiotensin-converting enzyme inhibitors (87.27% *vs* 51.32%, *p*<0.0001), enoxaparin (77.1% *vs* 72.2%, *p*>0.0001), and statins (95.1% *vs* 75.2%, *p*<0.0001), as well as in creatinine (1.47 mg/dL *vs* 2.09 mg/dL, *p*=0.03), ejection fraction (49.77% *vs* 43.14%, *p*<0.0001), tabagism (30.41% *vs* 38.71%, *p*=0.001), age (59 *vs* 70 years, *p*=0.03) and previous coronary artery bypass graft (14% *vs* 11%, *p*=0.003) between groups I and II (Table 1).

Multivariate analysis results are presented in Table 2, which describes the differences between groups I and II regarding the incidences of death (2.67% *vs* 9.09%, OR=0.35, *p*=0.02) and MACE (11% *vs* 29.5%, OR=4.55, *p*=0.02). There were no differences in the incidences of cardiogenic shock (3.5% *vs* 9.4%, OR=0.57, *p*=0.1) or sustained ventricular arrhythmia (0.7% *vs* 3.8%, OR=1.24, *p*=0.23) between the two groups (Table 2).

## DISCUSSION

In this study, we acquired results that differ from recently reported data. We found that the use of beta-blockers within the first 24 hours of ACS onset decreased in-hospital mortality and MACE. In contrast with other findings, the use of oral beta-blockers tended to reduce the incidence of cardiogenic shock.

The issue of whether to use beta-blockers in AMI has been discussed for more than 30 years. In 1981, Hjalmarson et al. 12 compared the effect of metoprolol on mortality in 1,395 patients with AMI; the metoprolol treatment began as soon after admission as possible and continued for 90 days. The mortality rates were 8.9% in the placebo group and 5.7% in the metoprolol group, resulting in an overall mortality reduction of 36% (*p*<0.03) 12. Similar results were observed in our study only when oral beta-blockers were administered.

Similarly, in the CAPRICORN trial, 1,959 patients with AMI and a left-ventricular ejection fraction of less than or equal to 40% were randomly assigned to receive oral carvedilol or placebo with follow-up until the requisite number of primary endpoints had occurred. Although there was no difference between the groups regarding the primary endpoint (all-cause mortality or hospital admission for cardiovascular problems), all-cause mortality was lower in the carvedilol group than the placebo group (12% *vs* 15%, 0.77 [0.60–0.98], *p*=0.03). In-hospital differences were not analyzed, and only 46% of the patients underwent percutaneous coronary intervention (PCI) or thrombolysis 13. These results are also concordant with our study, although we did not follow patients after discharge, and our study included patients with an ejection fraction greater than 40%.

A study published in 2014 evaluated 26,793 patients after undergoing a first cardiovascular event (ACS or coronary revascularization). Over an average of 3.7 years of follow-up, 6,968 patients experienced AMI or died. The use of beta-blockers was associated with reductions in mortality (HR=0.90; 95% confidence limits [CL]: 0.84 - 0.96) and death or AMI (HR=0.92; CL: 0.87 to 0.97). However, although the referenced study was recently published, the authors did not include information regarding the rates of PCI or in-hospital outcomes. Therefore, it was difficult to compare these findings with our results 14.

In another retrospective cohort study, the use of beta-blockers in 179 patients who experienced prehospital cardiac arrest was measured. The odds ratio for beta-blocker use among patients with cardiac arrest presenting as pulseless electrical activity *versus* ventricular fibrillation was 3.7 (95% CI 1.9—7.2), which indicates that a relationship exists between beta-blocker use and arrest rhythms 15. These findings were related to results from other trials showing a reduction in sustained ventricular arrhythmias with beta-blocker use after AMI and are in agreement with our results 7,8,16,17. Although the differences identified in our study were not significant, potentially due to the low number of included patients, there was a clear trend correlating the use of beta-blockers with a reduction in sustained ventricular arrhythmia. The most interesting finding is that the benefit of beta-blocker use was not associated with long-term prognosis, as has been reported in many previous studies, but rather with in-hospital outcomes starting within 24 hours of admission. We also observed a clear trend towards a reduction in sustained ventricular arrhythmia with beta-blocker use, although the relationship was not significant.

In 2005, the COMMIT trial was published. This study included 45,852 patients treated within 24 hours of AMI (93% had STEMI or bundle branch block) who were randomized into intravenous metoprolol and placebo groups. Among the patients in the metoprolol group, approximately 9.4% experienced at least one event compared with 9.9% of the patients in the placebo group (*p*=0.1). The use of metoprolol was related to lower rates of reinfarction (2.0% *vs* 2.5%; *p*=0.001) and ventricular fibrillation (2.5% *vs* 3.0%; *p*=0.001). However, the incidence of cardiogenic shock was higher in the metoprolol group (5.0% *vs* 3.9%; *p*<0.00001). Considering these results, intravenous beta-blocker use should be delayed until the hemodynamic condition after AMI has stabilized. Although the results in the referenced study argued against the use of beta-blockers in AMI, the overall conclusion that adequate use of oral beta-blockers within the first 24 hours of admission is beneficial is similar to our own findings, although caution should be exercised regarding any contra-indications. Overall, we found that clinical benefits were associated with the above approach 9.

Another recent study investigated the effects of beta-blockers in patients with STEMI in the PCI era. During a follow-up period of approximately 48 months, mortality did not differ between patients with and without beta-blocker use (5.2% *vs* 6.2%, *p*=0.786). However, in patients with a high ischemic risk (defined by the GRACE risk score), beta-blocker treatment was associated with a significantly lower mortality. Inconsistent with the COMMIT trial, the referenced study did not indicate that beta-blocker use was associated with worse results in any outcome. However, the authors proposed the individualization of treatment, commenting that the implementation of beta-blocker therapy in the PCI era for discharged patients may need to be assessed based on the individual mortality risk 18.

Due to the debate over the use of beta-blockers for ACS in the reperfusion era, a review of randomized trials evaluating beta-blocker treatment of AMI was undertaken. The primary outcome was all-cause mortality, and trials were divided into reperfusion era or pre-reperfusion era. Approximately 102,003 patients were included. It was found that beta-blockers significantly reduced mortality (*p*=0.02) in the pre-reperfusion era, but not in the reperfusion era. The incidences of AMI and angina were reduced in both groups. However, in the reperfusion era, increases in the incidences of heart failure and cardiogenic shock were related to the use of beta-blockers. Based on these findings, the authors proposed that guidelines should reconsider the strength of recommendations for beta-blockers post AMI. It is important to note that the main results obtained in the reperfusion era have been gathered by the COMMIT trial. However, in a sensitivity analysis that excluded this trial, there was still no benefit of beta-blockers regarding mortality in the reperfusion era. The categorization of pre-reperfusion *vs* reperfusion era was not performed based on calendar years, as there was wide variability in the use of medication and reperfusion. In addition, the referenced study considered both oral and intravenous beta-blockers 3.

Our results indicate that the use of beta-blockers within the first 24 hours after ACS in the reperfusion era could decrease in-hospital mortality and MACE. Important factors related to this relationship were identified, such as the exclusion of intravenous beta-blockers and the inclusion of both STEMI and NSTEMI. Additionally, the reduced in-hospital mortality identified in the present work has not been widely reported in the literature, possibly because most previous studies have focused on a long-term follow-up period.

### Limitations

This study had some limitations. For example, the design was observational, and only a small number of patients were included. Additionally, many of the baseline characteristics of the patients with and without beta-blockers were different. Furthermore, we did not separate the analysis according to type of beta-blocker used. All medications used in patients with coronary disease were administered according to the preferences of the physician. The rationale behind which medications were administered was not described.

In patients with acute coronary syndrome who undergo early intervention, the use of oral beta-blockers within the first 24 hours of symptom onset reduced in-hospital mortality and the incidence of MACE without increasing the incidences of cardiogenic shock and sustained ventricular arrhythmia.

## AUTHOR CONTRIBUTIONS

Soeiro AM, de Barros e Silva PG, Roque EA and Soeiro MC were responsible for data collection. Bossa AS, Zullino CN, Simões AS and Okada MY were responsible for data inclusion. Leal TC, Serrano Jr CV and Oliveira Jr MT were responsible for manuscript revision.

## Figures and Tables

**Table 1:** Baseline characteristics of patients with or without the use of beta-blockers within the first 24 hours of hospital admission.

	Beta-blocker +	Beta-blocker -	*p*
Age (median)	59	70	0.03
Male (%)	63%	59%	0.13
Diabetes mellitus (%)	39%	33%	0.68
Hypertension (%)	77%	71%	0.12
Tabagism (%)	30%	39%	0.001
FH of CAD (%)	13%	11%	0.47
Dyslipidemia (%)	49%	43%	0.12
Heart failure (%)	9%	4%	0.105
Previous stroke (%)	5%	5%	0.51
Previous AMI (%)	33%	31%	0.15
Previous CABG (%)	14%	11%	0.003
Previous PCI (%)	19%	21%	0.06
Ht (%) (median)	41.2	41.8	0.18
Cr (mg/dL) (median)	1.47	2.09	0.03
Troponin (ng/dL) (higher)	2.46	3.12	0.7
SAP (mmHg) (median)	133.7	127	0.29
EF (%) (median)	49.8	43.1	< 0.0001
AAS (%)	99%	95%	0.1
Enoxaparin (%)	77%	72%	<0.0001
ACE inhibitor (%)	87%	51%	<0.0001
Statin (%)	95%	75%	<0.0001

**Legend:** FH=familial history; CAD=coronary artery disease; AMI=acute myocardial infarction; CABG=coronary artery bypass grafting; PCI=percutaneous coronary intervention; SAP=systolic arterial pressure; Ht=hematocrit; Cr=creatinine; EF=ejection fraction; ACE=angiotensin-converting enzyme.

**Table 2:** Multivariate analysis of in-hospital outcomes for patients with or without the use of beta-blockers within the first 24 hours of admission.

	Beta-blocker +	Beta-blocker -	*OR*	CI (95%)	*p*
Reinfarction	1.0%	0.9%	0.83	0.62 – 4.67	0.9
Cardiogenic shock	3.5%	9.4%	0.57	0.29 – 1.12	0.1
Bleeding	2.3%	6.0%	1.28	0.57 – 2.90	0.55
SVA	0.7%	3.8%	1.24	0.82 – 2.56	0.23
Stroke	0.8%	0.3%	0.81	0.14 – 8.42	0.9
Mortality	2.7%	9.1%	8.12	1.53 – 14.56	0.02
MACE	11%	29.5%	4.55	2.05 – 10.09	0.032

**Legend:** CI=confidence interval; SVA=sustained ventricular arrhythmia; MACE=major adverse cardiac events.
